# Short and Long-Term Outcomes of PSARP versus LAARP and Single versus Staged Repair for Infants with High-Type Anorectal Malformations: A Systematic Review and Meta-Analysis

**DOI:** 10.3390/children11030376

**Published:** 2024-03-21

**Authors:** Maria Enrica Miscia, Giuseppe Lauriti, Dacia Di Renzo, Valentina Cascini, Gabriele Lisi

**Affiliations:** 1 Department of Medicine and Aging Science, “G. d’Annunzio” University of Chieti-Pescara, 66100 Chieti, Italy; mariaenrica.miscia@unich.it (M.E.M.); gabriele.lisi@unich.it (G.L.); 2 Pediatric Surgery Unit, “Spirito Santo” Hospital of Pescara, 65122 Pescara, Italy; dacia.direnzo@asl.pe.it (D.D.R.); valentina.cascini@asl.pe.it (V.C.)

**Keywords:** anorectal malformations, laparoscopic-assisted procedure, single-stage procedure, systematic review, meta-analysis

## Abstract

Background: We aimed to compare among patients with high-type anorectal malformations (ARM): (i) short- and long-term outcomes of laparoscopic-assisted anorectoplasty (LAARP) compared to classic posterior sagittal anorectoplasty (PSARP) and (ii) the results of single-stage versus staged PSARP. Methods: Using a defined search strategy, two independent investigators systematically reviewed the English literature. PRISMA guidelines were followed, and meta-analysis was performed using RevMan5.3. Results: Of 567 abstracts screened, 7 papers have been included (254 pts; 121 PSARP, 133 LAARP) in the first systematic review and meta-analysis. The length of hospitalization was shortened in LAARP versus PSARP (10.9 versus 14.4 days; *p* < 0.0001). PSARP and LAARP were comparable in terms of early postoperative complications (28.9% versus 24.7%; *p* = ns) and rectal prolapse (21.6% versus 17.5%; *p* = ns). At long-term follow-up, the presence of voluntary bowel movements (74.0% versus 83.5%; *p* = ns) and the incidence of soiling (45.5% versus 47.6%; *p* = ns) were similar in both PSARP and LAARP. Six papers (297 pts) were included in the second systematic review, with three comparative studies included in the meta-analysis (247 pts; 117 one-stage, 130 staged procedures). No significant difference in terms of presence of voluntary bowel movements after single-stage versus staged procedures (72.6% versus 67.3%; *p* = ns) has been detected. Conclusions: LAARP seems to be a safe and effective procedure, showing short- and long-term outcomes similar to PSARP. One-stage PSARP could be a safe alternative to the classic three-stage procedure, even for those infants with high-type ARM. Further and larger comparative studies would be needed to corroborate these partial existing data.

## 1. Introduction

The management of anorectal malformations (ARMs) has changed over the last decades [1]. Since the introduction of the Peña’s posterior sagittal anorectoplasty (PSARP) in the 1980s, further advancements have been achieved in order to improve the functional outcomes [1,2,3,4,5].

The classic PSARP approach is a three-stage procedure, which starts with a colostomy creation, followed by the anorectoplasty, with the later closure of the colostomy usually after 3 months [1,5,6].

Continence after the classic PSARP procedure ranges from 2 to 84%, depending on the type of ARM, the grade of development of the musculature, and the sacral ratio. The range of continence decreased to 0–32% when analyzing only the high-type ARM [6,7,8].

In 2000, Georgeson described a laparoscopic-assisted anorectoplasty (LAARP), which seemed to improve the visualization of the recto-urinary fistula and the correct center of the rectum in the muscle complex without dissecting the muscles, thus promising better outcomes [1,2,3,8,9,10,11].

In recent years, a single-stage repair of ARMs has been proposed in order to reduce the number of surgeries and anesthesia, to reach the early restoration of the anatomy and physiology of the lower GI tract, and to avoid the colostomy creation, thus ideally ameliorating the outcomes of the classic PSARP procedure [2,4,5,6,7,12,13].

The one-stage procedure for infants with low ARMs has shown encouraging results [14,15]. However, little is known about the short- and the long-term outcomes of patients with high-type ARMs (i.e., recto-urethral, recto-vesical fistulas, and rectovaginal fistulas) [12].

Therefore, we aimed to compare the short- and long-term outcomes of male patients with high ARMs treated with LAARP or PSARP procedures. Moreover, we aimed to assess the feasibility of the one-stage procedure in the same cohort of patients, thus comparing the outcomes of the single-stage versus the staged procedures in infants with high-type ARMs.

## 2. Material and Methods

### 2.1. Data Sources and Study Selection

This study was registered on the international prospective register of systematic reviews PROSPERO (registration #CRD42022359940) (National Institute for Health Research) [16]. The systematic review was drafted according to the Preferred Reporting Items for Systematic Reviews and Meta-Analyses (PRISMA) statement [17].

A systematic review of the English literature was made using a defined search strategy (Table 1).

Two authors (MEM, GLa) individually screened different databases (PubMed, Cochrane Collaboration, Scopus, and Web of Science) in order to select those papers focusing on anorectal malformations published till November 2023. MeSH headings and keywords applied were “one stage PSARP”, “single stage PSARP”, “anorectal malformation AND stage” and “anorectal malformation repair”, “LAARP AND anorectal malformation” (Supplementary File S1). The same authors also screened the reference lists of those eligible studies in order to identify pertinent cross-references. Exclusion criteria were case reports, those case series with less than 10 patients, opinion articles, and experimental papers. Grey literature (i.e., reports, theses, conference proceedings, bibliographies, commercial documentations, and official documents not published commercially) were excluded. Full text manuscripts of theoretically suitable papers have been saved and autonomously considered for appropriateness by the same two authors (MEM, GLa).

All those papers (trials, cohort, and case-control) reporting at least one outcome of interest have been included, according to our PICO (Supplementary File S2). Moreover, we included in the meta-analysis all papers comparing the outcomes of LAARP versus PSARP procedures to repair high-type ARMs and/or the single versus staged repair in the same patients. In cases of overlapping cohorts of patients, the authors selected the paper with the largest number of children for each outcome. The divergence over the appropriateness of a particular paper was solved thanks to the discussion with a further investigator (GLi).

### 2.2. Statistical Analysis

Categorical variable rates were compared with Pearson’s chi-square test or the two-tailed Fisher exact probability test. When median and range have been reported, mean ± SD were estimated, as previously reported [18]. Meta-analysis was managed with RevMan 5.4 [19], with a random effects model. Risk ratio (RR) has been considered for categorical variables. Mean differences (MDs) have been selected in those continuous variables. Results were reported with 95% confidence intervals (CIs). Data are expressed as mean ± SD. I^2^ values were considered to assess homogeneity and quantify the dispersion of effect sizes. Biases among those studies included were assessed with the funnel plot. Quantitative and demographic data were compared using Fisher’s exact test and are expressed as number, percentage, or mean ± SD, using the RR and 95% CIs. A *p* < 0.05 was considered significant.

### 2.3. Quality Assessment

Two independent authors (DDR and VC) evaluated the risk of bias for individual papers, thanks to the methodological index for non-randomized studies (MINORS) [20]. Dissimilarities between them were solved through debate and agreement with a further investigator (GLa). The score for this 12-item index ranges between 0 and 24 points. The validated “gold standard” cut-off was 19.8 points. With regards to the quality for each outcome, authors graded the quality of evidence thanks to the Grading of Recommendations Assessment, Development and Evaluation (GRADE) methodology [21]. The quality of evidence has been ranked as high, moderate, low, and very low for all outcomes. Observational studies were assessed with a low quality of evidence. The quality of evidence has been decreased in case of risk of bias, inconsistency, indirectness imprecision, and publication issues. The MINORS index has been assumed to assess the risk of bias in comparative studies. Inconsistency has been judged according to heterogeneity, and the I^2^ value was applied to evaluate heterogeneity. I^2^ values of 0–40, 30–60, 50–90, and 75–100% were considered as low, moderate, substantial, and considerable heterogeneity, respectively. Imprecision was evaluated using optimal information size (OIS), which was based on 25% relative risk reduction, 0.05 of α error, and 0.20 of β error [22].

## 3. Results

**Figure 1:**
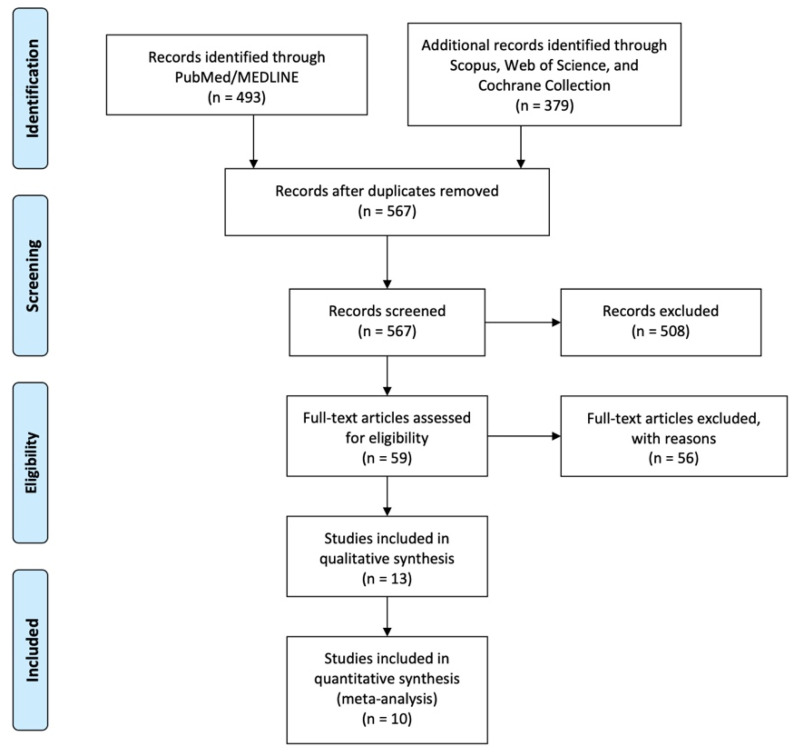
Diagram of workflow in the systematic review and meta-analysis, adapted from prisma-statement.org (accessed on 31 January 2024) [17].

Of 567 abstracts and titles screened, we included (Figure 1, Table 2):

1. Seven papers (254 pts; 121 PSARP, 133 LAARP) in the systematic review and in the meta-analysis to compare the postoperative outcomes of PSARP and LAARP in infants with high-type ARMs [1,2,3,8,9,10,11]. Only one randomized controlled study was included [10]. One further study prospectively evaluated the outcomes of the patients [1]. All other studies were retrospective.
2. Six papers (297 pts) in the systematic review and three comparative studies (247 pts; 117 one-stage, 130 staged procedures) in the meta-analysis to compare the results of the single- versus the three-stage correction of high ARMs [4,5,6,7,12,13]. All studies were retrospective.

### 3.1. PSARP versus LAARP Procedures

Systematic Review: Among the 254 patients included, 164 (64.5%) had an ARM with a recto-urethral fistula (77 in the PSARP group and 85 in the LAARP group), 54 (21.3%) had a recto-vesical fistula (25 in the PSARP group and 29 in the LAARP group), 7 (2.8%) had a rectovaginal fistula (3 in the PSARP group and 4 in the LAARP group), and 16 (6.3%) had no fistula (10 undergoing PSARP and 6 LAARP procedure). In 13 (5.1%) patients, the type of fistula was not specified.

**Figure 2:**
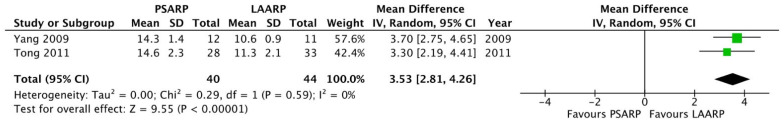
Forest plot comparison of length of hospitalization in the LAARP versus the PSARP groups [2,10].

**Figure 3:**
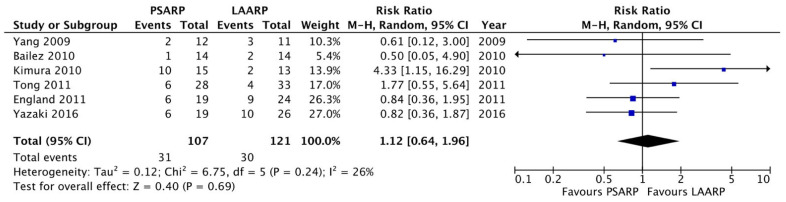
Forest plot comparison of early postoperative complication in the LAARP versus the PSARP groups [1,2,3,8,9,10].

**Figure 4:**
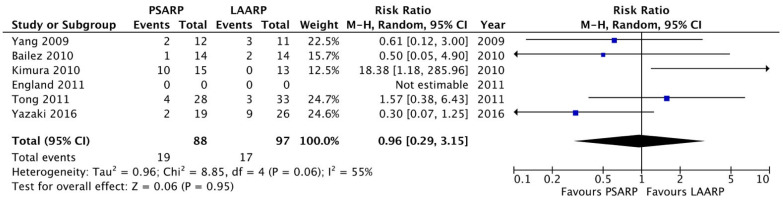
Forest plot comparison of rectal prolapse in the LAARP versus the PSARP groups [1,2,3,8,9,10].

**Figure 5:**
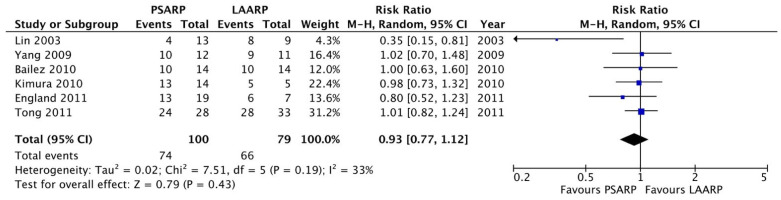
Forest plot comparison of the presence of voluntary bowel movements in the LAARP versus the PSARP groups [1,2,3,8,10,11].

**Figure 6:**
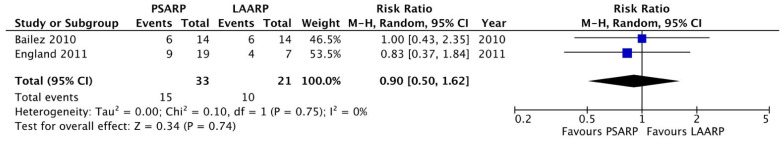
Forest plot comparison of the incidence of soiling in the LAARP versus the PSARP groups [1,8].

Meta-analysis: We found a significant shorter length of hospitalization in the LAARP versus the PSARP groups (10.9 ± 0.5 versus 14.4 ± 0.2 days, respectively; *p* < 0.0001; Figure 2). The two procedures were comparable in terms of early postoperative complications (PSARP 31/107 pts, 28.9% versus LAARP 30/121 pts, 24.7%; *p* = ns, Figure 3) and rectal prolapse (PSARP 19/88 pts, 21.6% versus LAARP 17/97 pts, 17.5%; *p* = ns, Figure 4). When analyzing the long-term results, we also found the same presence of voluntary bowel movements (74/100 pts; 74.0% versus 66/79 pts; 83.5%; *p* = ns, Figure 5) and a similar incidence of soiling (15/33 pts; 45.5% versus 10/21 pts; 47.6%; *p* = ns, Figure 6) in both PSARP and LAARP (Table 3).

### 3.2. Single-Stage versus Staged Procedures

Systematic Review: Among the 297 patients included in the study, 167/297 (56.2%) patients underwent a one-stage repair of the fistula and 130 (43.8%) a staged procedure. A total of 104/297 (35.1%) patients had a recto-urethral fistula and 1/297 (0.3%) patients had a recto-vesical fistula. No data were available regarding the type of fistula for the remaining 192 patients (64.6%).

**Figure 7:**
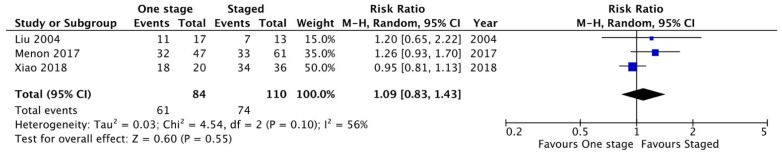
Forest plot comparison of the presence of voluntary bowel movements after the single stage versus staged procedures [4,7,13].

Meta-analysis: We found the same incidence in terms of presence of voluntary bowel movements after the single-stage versus staged procedures (61/84, 72.6% versus 74/110, 67.3%; *p* = ns, Figure 7). Only the study by Xiao et al. [4] compared the post-operative complications and the incidence of constipation and soiling after the single versus staged repair: no differences were found between the two groups (*p* = ns) (Table 4).

## 4. Discussion

Anorectal malformations have an incidence of 1:4000–5000 livebirths [2,23].

The aims of surgical repair of ARMs are to divide the rectum from the urinary tract, to correctly place the rectum at the center of the muscle complex, and to achieve voluntary bowel movements and continence [1]. However, irrespectively from the procedure used, continence is also dependent on a well-developed sacrum (sacral ratio > 0.6) and a good perineal musculature [6]. Moreover, an early stimulation of a brain-defecatory reflex is of utmost importance, since during the early neonatal period, the neuronal networks responsible for the reflex formation develop [5,6,7,12].

The gold standard treatment to repair ARMs is the classic PSARP, firstly described by Peña in the 1980s [6,10]. This approach is based on three stages, with a stoma creation at first, followed by the anorectoplasty, and finally with the stoma closure [4,6].

In the early 2000s, Georgeson introduced the use of laparoscopy for the ARM correction [2,8]. The LAARP has several advantages over the classic PSARP, thanks to a better and direct visualization of the fistula, with an improved placement of the rectum at the center of the perineal muscle complex. Moreover, LAARP guarantees no or minimal dissection of the muscle fibers, thus reducing the risk of nerve injury [2,3]. The less manipulation of the muscles and nerves may be responsible for the better outcomes of the LAARP over the PSARP. In fact, some authors have reported a higher incidence of the recto-anal inhibitory reflex (RIRA) after LAARP when compared to PSARP, with better outcomes in terms of fecal continence [1,2,9,11]. Other authors have reported comparable functional outcomes among the two groups [3,10]. Nevertheless, many studies agreed that the outcomes improve over time, especially if a post-operative bowel management program has been planned at follow-up [8,9,23].

The main complication after LAARP is the rectal mucosal prolapse, which is reported in 9–46% of patients [9]. However, this complication could be avoided by performing a minimal dissection of the mesorectum and/or performing a rectopexy [2,3].

However, the majority of the studies did not differentiate among the types of ARMs, thus leading to a bias linked to the different prognosis of the different types of ARMs themselves.

In our study, we have focused on newborns with high-type ARMs (i.e., recto-urethral and recto-vesical fistulas, and rectovaginal fistulas) and we have found a shorter hospitalization after LAARP compared to the PSARP procedure, leading to a quicker recovery. Moreover, we did not find a higher incidence of rectal prolapse among the LAARP group, thus suggesting that it is not as frequent as it has been postulated. In terms of early and late post-operative outcomes, we found that the presence of voluntary bowel movements and the incidence of soiling was not different among patients undergoing LAARP or PSARP repair of ARMs. All these data seem to suggest that LAARP is a valid alternative to PSARP.

The LAARP was also used to perform a single-stage repair of the ARM, without the colostomy formation [2]. The rationale for a stoma creation is to allow the correct location of the fistula by performing a late cologram, to decompress the colon, to avoid infections, and to protect the anastomosis from stool passage [4]. However, colostomy seems not to be free of complications. In fact, up to 74% of patients have reported developing stoma-related complications (such as prolapse, retraction, and adhesions). Moreover, many patients with a colostomy have been lost at long-term follow-up, especially in developing countries [6,12,13]. Furthermore, the presence of a colostomy requires several surgeries in order to close it [5,7,12].

Therefore, in order to avoid the problems related to staged procedures, some authors started to perform a single-stage approach in well-selected patients (i.e., those without major cardiac anomalies or sepsis) [24]. The advantages of a single-stage approach were the avoidance of multiple surgeries and anesthesia, the absence of a colostomy (and colostomy-related complications), and an early restoring of the anatomy, thus allowing an early training of the perineal musculature [4,5,6,7,12,13]. The early restoration of the bowel continuity has been reported to be especially important for a precocious creation of the neuronal networks responsible for the brain-defecation reflex, thus ideally leading to better outcomes in terms of continence [4,5,6,7,12,13]. Nonetheless, the single-stage procedure would also reduce the burden related to the psychological familial aspect of repeated surgeries and long follow-up [12].

The main concerns related to the single-stage procedure are the unawareness of the fistula location, the risk of secondary infections due to the presence of a colon dilated with meconium, and the lack of follow-up data [6,12,13,14]. However, these aspects do not seem to emerge from our review. To avoid the problems related to the level of the fistula, some authors have suggested performing a preoperative cystoscopy in order to visualize the fistula and even to perform a trans-fistula enema [12,25]; some other authors have suggested a transperineal evacuation of the meconium in order to obtain colon decompression, thus reducing the risk of infections [6,12].

To date, few reports are available regarding the short- and long-term outcomes of the one-stage correction of infants with high ARMs. As a matter of fact, in our study, we could include only three comparative papers. Nonetheless, we found that the presence of voluntary bowel movements did not differ among patients after single-stage or staged repair. Moreover, the reported incidence of soiling and constipation was comparable between the two groups. These data partially confirm and reinforce the idea that a single-stage repair of patients with high-type ARMs could be a safe and feasible option in well-selected cases.

### Limitations of the Study

There are few limitations in the present meta-analysis. All but one paper were retrospective, as already mentioned. This aspect could produce selected bias. Moreover, not one study specified a sample-size calculation. As predictable, a blinded evaluation of impartial outcomes was not feasible. Furthermore, the number of children reported were generally not considerable. Both the LAARP and single-stage technique could be related to the volume of procedures. Therefore, those centers with a high volume of patients could present improved short- and long-term results compared to low-volume centers. Additionally, none of the included papers reported the loss of patients during the follow-up. Consequently, in our study, none of the included papers obtained the gold standard cut-off on MINORS (Table 5).

Looking at the GRADE assessment, our meta-analysis reached a low quality of evidence on the incidence of complications and the presence of voluntary bowel movements, comparing LAARP versus PSARP, and very low with regards the other outcomes (Table 6). Either the reduced quantity of patients or the significant heterogeneousness of the records could produce potential bias.

However, the present meta-analysis obtained a reliable result when evaluated in duplicate (DDR and VC), thanks to A Measurement Tool to Assess Systematic Reviews (AMSTAR, Supplementary File S3) [26].

The PRISMA checklist has been lastly finalized (Supplementary File S4).

## 5. Conclusions

The management of ARMs is in continuous evolution in order to achieve better short- and long-term outcomes and to improve the quality of life of these children.

Even if few data have been published with regards to the management of infants with high-type ARMs, it seems that the three-stage LAARP might be a safe and valid alternative to the three-stage PSARP, with a quicker recovery of the patients. Moreover, it seems that the single-stage anorectoplasty (including the laparoscopic-assisted procedure) might be a safe and feasible alternative, which could reduce the problems related to the longer three-stage PSARP with the stoma creation and might allow an early restoration of the brain-defecatory axis, which is of utmost importance for achieving a good continence as well as better defecatory behaviors.

However, the reported data are still limited to the best of our knowledge, thus further and larger studies would be needed to corroborate and reinforce these preliminary findings. Up to now, despite of any approach used in the surgical treatment of high-type ARMs, the results seem not to be improved.

## Figures and Tables

**Table 1:** Inclusion criteria of the systematic review.

Publication	
Language	English
Time period	January 1984–November 2023
Subject	Human studies
Study type	Retrospective
	Prospective
	Case-control
	Cohort
Excluded	Case-report
	Case series (<10 patients)
	Editorials
	Letters
	Grey Literature
Keywords	anorectal malformation
	posterior sagittal anorectoplasty
	one stage
	single stage
	laparoscopic-assisted anorectoplasty

**Table 2:** Demographic data on studies included in meta-analysis.

3-Stage PSARP vs. 1-Stage					
Paper	Pts	BW	GA	Age at Surgery	Associated Anomalies
Xiao [4]	56 3-stage 36 1-stage 20	NR	NR	3-stage 4.9 ± 1.2 (3–7) months 1-stage 39.8 ± 8.1 (30–52) hours	3-stage: 15 cardiac anomalies 5 VUR 5 undescended testes 2 hypospadias 6 hydronephrosis 4 spina bifida occulta 5 partial sacral agenesis 1 tethered cord 1-stage: 10 cardiac anomalies 2 VUR 1 undescended testis 1 hypospadias 2 hydronephrosis 2 spina bifida occulta 4 partial sacral agenesis
Leva [5]	23 3-stage 4 1-stage 19	3-stage 2.9–3.4 kg 1-stage 2.4–3.5 kg	3-stage at term 1-stage 35–41 w		3-stage: none 1-stage: 2 ASD 1 VSD 1 pulmonary stenosis 4 VUR 3 hypospadias 1 MCKD 1 ectopic kidney
Nagdeve [6]	NR	NR	NR	NR	NR
Menon [7]	NR	NR	NR	NR	NR
Agrawal [12]	15 1 stage	2.5 ± 0.4 kg	36 ± 5 weeks		
Liu [13]	113 3-stage 43 1-stage 65	NR	NR	NR	3-stage: 2 hypospadias 3 sacral anomalies 3 cryptorchidism 3 inguinal hernia 3 trisomy21 1-stage: 5 sacral anomalies 1 hypospadia 1 cryptorchidism
**PSARP vs. LAARP**					
England [1]	53 PSARP 19 LAARP 24	PSARP NR LAARP 3 (2–3) kg median	NR	PSARP 8 (4–39) months (median) LAARP 7 (2–15) months (median)	PSARP NR LAARP 3 VSD 1 pulmonary stenosis 1 MCKD 1 hydronephrosis 2 renal ectopia 1 VUR 3 renal agenesis 1 absent thumb 1 rib fusion 1 dysmorphism
Tong [2]	61 PSARP 28 LAARP 33	NR	NR	PSARP 4.9 (3–11) months LAARP 5.3 (3–10) months	
Kimura [3]	28 PSARP 15 LAARP 13	NR	NR	NR	NR
Bailez [8]	32 PSARP 15 LAARP 17	NR	NR	PSARP Rectoprostatic f. 29.2 months Rectovesical f. 25.7 months LAARP Rectoprostatic f. 22 months Rectovesical f. 37.5 months	25/32 (78%) pts-associated anomalies
Yazaki [9]	45 PSARP 19 LAARP 26	NR	NR	PSARP Rectoprostatic f. 4 ± 3.5 months Rectobulbar f. 8.2 ± 5.1 months LAARP Rectoprostatic f. 7.6 ± 3 months Rectobulbar f. 8.1 ± 4 months	NR
Yang [10]	23 PSARP 12 LAARP 11	NR	NR	NR	NR
Lin [11]	16 PSARP 10 LAARP 6	NR	NR	NR	PSARP 2 hypospadias 1 hydronephrosis 1 trisomy 21 LAARP 1 cardiac anomaly 1 hypospadias 1 trisomy 21

BW: birth weight, GA: gestational age, NR: not reported.

**Table 3:** Summary of the results of the comparison of PSARP versus LAARP. *p* is statistically significant when <0.05.

Outcomes	PSARP	LAARP	*p*-Value
Length of hospitalization, days	14.4 ± 0.2	10.9 ± 0.5	<0.0001
Post-operative complications, *n* of pts (%)	31/107 (28.9)	30/121 (24.7)	0.69
Rectal prolapse, *n* of pts (%)	19/88 (21.6)	17/97 (17.5)	0.95
Voluntary bowel movements, *n* of pts (%)	74/100 (74)	66/79 (83.5)	0.43
Soiling, *n* of pts (%)	15/33 (45.5)	10/21 (47.6)	0.74

**Table 4:** Summary of the results of the comparison of single-stage versus staged correction of ARMs. *p* is statistically significant when <0.05.

Outcomes	Single Stage	Staged	*p*-Value
Voluntary bowel movements, *n* of pts (%)	61/84 (72.6)	74/110 (67.3)	0.55
Soiling, *n* of pts (%)	4/20 (20)	6/36 (16)	1
Constipation, *n* of pts (%)	6/20 (30)	9/36 (25)	1

**Table 5:** Risk of bias assessment for individual studies, adapted from the methodological index for nonrandomized studies (MINORS) [20].

Item	England [1]	Tong [2]	Kimura [3]	Xiao [4]	Menon [7]	Bailez [8]	Yazaki [9]	Yang [10]	Lin [11]	Liu [13]
1. A clearly stated aim	2	2	2	2	2	2	2	2	2	2
2. Inclusion of consecutive patients	2	2	2	2	2	2	2	2	2	2
3. Prospective collection of data	2	0	0	0	0	0	0	2	0	0
4. Endpoints appropriate to the aim of the study	2	2	2	2	2	2	2	2	2	2
5. Unbiased assessment of the study endpoint	0	0	0	0	0	0	0	0	0	0
6. Follow-up period appropriate to the aim of the study	2	2	2	2	1	2	2	2	1	2
7. Loss to follow-up less than 5%	0	0	0	0	0	0	0	0	0	0
8. Prospective calculation of the study size	0	0	0	0	0	0	0	0	0	0
9. An adequate control group	2	2	2	2	2	2	2	2	2	2
10. Contemporary groups	2	2	2	2	2	2	2	2	2	2
11. Baseline equivalence of groups	2	2	2	2	1	2	2	2	2	2
12. Adequate statistical analyses	2	2	2	2	2	2	2	2	2	2
Total score	18	16	16	16	14	16	16	18	15	16

**Table 6:** GRADE evidence profile for the present meta-analysis, adapted from gradepro.org (accessed on 31 January 2024) [21].

Quality Assessment							No. of Patients		Effect		Quality
No. of Studies	Study Design	Risk of Bias	Inconsistency	Indirectness	Imprecision	Other Considerations	Cases	Controls	Relative (95% CI)	Absolute (95% CI)	
LOS in LAARP versus PSARP							LAARP	PSARP			
2	OS	Moderate ^a^	Low	Not serious	Serious ^b^	None	44	40	---	MD 3.53 lower (from 2.81 to 4.26 lower)	⊗OOOVERY LOW
Complication in LAARP versus PSARP							LAARP	PSARP			
6	OS	Moderate ^a^	Low	Not serious	Serious ^b^	None	30/121 (24.8%)	31/107 (28.9%)	RR 1.12 (0.64, 1.96)	41 fewer per 1000 (from 328 fewer to 123 more)	⊗⊗OOLOW
Rectal prolapse in LAARP versus PSARP							LAARP	PSARP			
6	OS	Moderate ^a^	Moderate	Not serious	Serious ^b^	None	17/97 (17.5%)	19/88 (21.6%)	RR 0.96 (0.29, 3.15)	41 fewer per 1000 (from 2204 fewer to 728 more)	⊗OOOVERY LOW
Voluntary bowel movements in LAARP versus PSARP							LAARP	PSARP			
6	OS	Moderate ^a^	Moderate	Not serious	Serious ^b^	None	66/79 (83.5%)	74/100 (74.0%)	RR 0.93 (0.77, 1.12)	95 more per 1000 (from 163 fewer to 312 more)	⊗⊗OOLOW
Soiling in LAARP versus PSARP							LAARP	PSARP			
2	OS	Moderate ^a^	Low	Not serious	Serious ^b^	None	10/21 (47.6%)	15/33 (45.5%)	RR 0.90 (0.50, 1.62)	21 more per 1000 (from 130 fewer to 105 more)	⊗OOOVERY LOW
Voluntary bowel movements in one-stage versus staged PSARP							One stage	Staged			
3	OS	Moderate ^a^	Moderate	Not serious	Serious ^b^	None	61/84 (72.6%)	74/110 (67.3%)	RR 1.09 (0.83, 1.43)	53 more per 1000 (from 100 fewer to 253 more)	⊗OOOVERY LOW

LOS: length of Hospital stay; LAARP: laparoscopic-assisted ano-rectoplasty; PSARP: posterior sagittal ano-rectoplasty. ^a^ Bias due to possible confounding; ^b^ OIS not met GRADE Working Group grades of evidence. High quality: Further research is very unlikely to change our confidence in the estimate of effect. Moderate quality: Further research is likely to have an important impact on our confidence in the estimate of effect and may change the estimate. ⊗⊗ Low quality: Further research is very likely to have an important impact on our confidence in the estimate of effect and is likely to change the estimate. ⊗ Very low quality: We are very uncertain about the estimate.

## Supplementary Materials

The following Supporting Information can be downloaded at: https://www.mdpi.com/article/10.3390/children11030376/s1, Supplementary File S1: Search strategy; Supplementary File S2: PICO; Supplementary File S3: AMSTAR criteria [26] for the present systematic reviews and meta-analysis assessed by two authors; Supplementary File S4: PRISMA checklist.

## References

[1] England R.J., Warren S.L., Bezuidenhout L., Numanoglu A., Millar A.J. (2012). Laparoscopic repair of anorectal malformations at the Red Cross War Memorial Children’s Hospital: Taking stock. J. Pediatr. Surg..

[2] Tong Q.-S., Tang S.-T., Pu J.-R., Mao Y.-Z., Wang Y., Li S.-W., Cao Q.-Q., Ruan Q.-L. (2011). Laparoscopically assisted anorectal pull-through for high imperforate anus in infants: Intermediate results. J. Pediatr. Surg..

[3] Kimura O., Iwai N., Sasaki Y., Tsuda T., Deguchi E., Ono S., Furukawa T. (2010). Laparoscopic versus open abdominoperineal rectoplasty for infants with high-type anorectal malformation. J. Pediatr. Surg..

[4] Xiao H., Huang R., Chen L. (2018). The midterm outcomes of 1-stage versus 3-stage laparoscopic-assisted anorectoplasty in anorectal malformations with rectoprostatic fistula and rectobulbar fistula: A retrospective cohort study. Medicine.

[5] Leva E., Macchini F., Arnoldi R., Di Cesare A., Gentilino V., Fumagalli M., Mosca F., Bhuiyan A., Torricelli M., Banu T. (2013). Single-stage surgical correction of anorectal malformation associated with rectourinary fistula in male neonates. J. Neonatal Surg..

[6] Nagdeve N., Naik H., Bhingare P. (2011). Neonatal posterior sagittal anorectoplasty for a subset of males with high anorectal malformations. J. Indian Assoc. Pediatr. Surg..

[7] Menon P., Rao K.L.N., Sinha A.K., Lokesha K., Samujh R., Mahajan J.K., Kanojia R.P., Bawa M. (2017). Anorectal Malformations in Males: Pros and Cons of Neonatal versus Staged Reconstruction for High and Intermediate Varieties. J. Indian Assoc. Pediatr. Surg..

[8] Bailez M.M., Cuenca E.S., Mauri V., Solana J., Di Benedetto V. (2011). Outcome of males with high anorectal malformations treated with laparoscopic-assisted anorectal pull-through: Preliminary results of a comparative study with the open approach in a single institution. J. Pediatr. Surg..

[9] Yazaki Y., Koga H., Ochi T., Okawada M., Doi T., Lane G.J., Yamataka A. (2016). Surgical management of recto-prostatic and recto-bulbar anorectal malformations. Pediatr. Surg. Int..

[10] Yang J., Zhang W., Feng J., Guo X., Wang G., Weng Y., Sun X., Yu D. (2009). Comparison of clinical outcomes and anorectal manometry in patients with congenital anorectal malformations treated with posterior sagittal anorectoplasty and laparoscopically assisted anorectal pull through. J. Pediatr. Surg..

[11] Lin C.L., Wong KK Y., Lan LC L., Chen C.C., Tam P.K.H. (2003). Earlier appearance and higher incidence of the rectoanal relaxation reflex in patients with imperforate anus repaired with laparoscopically assisted anorec- toplasty. Surg. Endosc..

[12] Agrawal V., Sharma D., Tiwari A., Mishra R., Acharya H. (2020). Transperineal Intracath Meconiolysis and Evacuation Technique of “Distended” Bowel Evacuation for One-Stage Laparoscopic Anorectoplasty for High Anorectal Malformations in Males. J. Laparoendosc. Adv. Surg. Tech. A.

[13] Liu G., Yuan J., Geng J., Wang C., Li T. (2004). The treatment of high and intermediate anorectal malformations: One stage or three procedures?. J. Pediatr. Surg..

[14] Lauriti G., Di Renzo D., Chiesa P.L., Zani A., Pierro A. (2019). One-stage repair of anorectal malformations in females with vestibular fistula: A systematic review and meta-analysis. Pediatr. Surg. Int..

[15] Hartford L., Brisighelli G., Gabler T., Westgarth-Taylor C. (2022). Single-stage procedures for anorectal malformations: A systematic review and meta-analysis. J. Pediatr. Surg..

[16] PROSPERO International Prospective Register of Systematic Reviews. https://www.crd.york.ac.uk/prospero.

[17] Moher D., Liberati A., Tetzlaff J., Altman D.G., The PRISMA Group (2009). Preferred reporting items for systematic reviews and meta-analyses: The PRISMA statement. PLoS Med..

[18] Hozo S.P., Djulbegovic B., Hozo I. (2005). Estimating the mean and variance from the median, range, and the size of a sample. BMC Med. Res. Methodol..

[19] Review Manager (RevMan) (2014). The Nordic Cochrane Centre.

[20] Slim K., Nini E., Forestier D., Kwiatkowski F., Panis Y., Chipponi J. (2003). Methodological index for non-randomized studies (MINORS): Development and validation of a new instrument. ANZ J. Surg..

[21] Guyatt G.H., Oxman A.D., Vist G.E., Kunz R., Falck-Ytter Y., Alonso-Coello P., Schünemann H.J. (2008). GRADE: An emerging consensus on rating quality of evidence and strength of recommendations. BMJ.

[22] Dupont W.D., Plummer W.D. (1990). Power and sample size calculations: A review and computer program. Control. Clin. Trials.

[23] Leung M.W.Y., Wong B.P.Y., Leung A.K.P., Cho J.S.Y., Leung E.T.Y., Chao N.S.Y., Chung K.W., Kwok W.K., Liu K.K.W. (2006). Electrical stimulation and biofeedback exercise of pelvic floor muscle for children with faecal incontinence after surgery for anorectal malformation. Pediatr. Surg. Int..

[24] Zheng S., Xiao X., Huang Y. (2008). Single-stage correction of imperforate anus with a rectourethral or a rectovestibula fistula by semi-posterior sagittal anorectoplasty. Pediatr. Surg. Int..

[25] Li L., Yu Q., Huang L. (2003). One-stage correction of high imperforate anus with laparoscopy-assisted anorectoplasty. Chin. J. Minim. Invasive Surg..

[26] Shea B.J., Grimshaw J.M., Wells G.A., Boers M., Andersson N., Hamel C., Porter A.C., Tugwell P., Moher D., Bouter L.M. (2007). Development of AMSTAR: A measurement tool to assess the methodological quality of systematic reviews. BMC Med. Res. Methodol..

