# Supplementary material for: Short and Long-Term Outcomes of PSARP versus LAARP and Single versus Staged Repair for Infants with High-Type Anorectal Malformations: A Systematic Review and Meta-Analysis
Source: Children (Basel). 2024 Mar 21;11(3):376. doi: 10.3390/children11030376 (PMC10968977; doi:10.3390/children11030376)
Supplement: Supplementary file 1 [file children-11-00376-s001.zip › Supplementary files/Supplementary file S1.doc]

**Supplementary file S1:** Search strategy.

**PubMed/MEDLINE**

1. (one stage OR single stage).mp.
2. (posterior sagittal anorectoplasty*adj2).mp.
3. (laparoscopic-assisted anorectoplasty*adj2).mp.
4. (anorectal malformation*adj2).mp.
5. 1 AND 2 AND 4
6. 3 AND 4

**Scopus**

TITLE-ABS-KEY ( ( one stage ) OR ( single stage ) AND ( posterior sagittal anorectoplasty ) AND ( anorectal malformation ) )

TITLE-ABS-KEY ( ( laparoscopic-assisted anorectoplasty ) AND ( anorectal malformation ) )

**Cochrane Collaboration**

1. MeSH descriptor : one stage, single stage, posterior sagittal anorectoplasty, anorectal malformation
2. MeSH descriptor : laparoscopic-assisted anorectoplasty, anorectal malformation
3. Explode all trees

**Web of Science**

TOPIC ( ( one stage ) OR ( single stage ) AND ( posterior sagittal anorectoplasty ) AND ( anorectal malformation ) )

TOPIC ( ( laparoscopic-assisted anorectoplasty ) AND ( anorectal malformation ) )
