# Supplementary material for: Short and Long-Term Outcomes of PSARP versus LAARP and Single versus Staged Repair for Infants with High-Type Anorectal Malformations: A Systematic Review and Meta-Analysis
Source: Children (Basel). 2024 Mar 21;11(3):376. doi: 10.3390/children11030376 (PMC10968977; doi:10.3390/children11030376)
Supplement: Supplementary file 1 [file children-11-00376-s001.zip › Supplementary files/Supplementary file S2.doc]

**Supplementary file S2:** PICO.

**Population**: neonates with high Anorectal Malformation (ARM)

**Intervention**: surgical repair of ARM

**Comparison**: 1) Laparoscopic-Assisted Ano-Recto-Plasty (LAARP) *versus*

Posterior-Sagittal-Ano-Recto-Plasty (PSARP)

2) Single stage *versus* Staged Procedure

**Outcomes**: Early Post-operative complications

Length of hospital Stay

Rectal Prolapse

Long-term postoperative complications

Voluntary Bowel Movements

Soiling

|  | Inclusion Criteria | Exclusion Criteria |
| --- | --- | --- |
| Study design | Case/control studies  Randomized Control Trials  Prospective studies  Retrospective studies | Reviews  Case series <10 pts  Editorials  Grey Literature |
| Population | Neonates with high ARM | Any other type of ARM |
| Intervention | Surgical correction of ARM |  |
| Comparison | 1) PSARP *versus* LAARP 2) Staged *versus* Single Stage | Any other procedure |
