# Supplementary material for: Short and Long-Term Outcomes of PSARP versus LAARP and Single versus Staged Repair for Infants with High-Type Anorectal Malformations: A Systematic Review and Meta-Analysis
Source: Children (Basel). 2024 Mar 21;11(3):376. doi: 10.3390/children11030376 (PMC10968977; doi:10.3390/children11030376)
Supplement: Supplementary file 1 [file children-11-00376-s001.zip › Supplementary files/Supplementary file S3.doc]

**Supplementary file S3: AMSTAR criteria [26] for the present systematic reviews and meta-analysis assessed by two authors.**

| Item | DDR | VC |
| --- | --- | --- |
| 1. Was an 'a priori' design provided? | 1 | 1 |
| 2. Was there duplicate study selection and data extraction? | 1 | 1 |
| 3. Was a comprehensive literature search performed? | 1 | 1 |
| 4. Was the status of publication (i.e. grey literature) used as an inclusion criterion? | 1 | 1 |
| 5. Was a list of studies (included and excluded) provided? | 0 | 0 |
| 6. Were the characteristics of the included studies provided? | 1 | 1 |
| 7. Was the scientific quality of the included studies assessed and documented? | 1 | 1 |
| 8. Was the quality of the included studies used appropriately in formulating conclusions? | 0 | 1 |
| 9. Were the methods used to combine the findings of studies appropriate? | 1 | 1 |
| 10. Was the likelihood of publication bias assessed? | 1 | 1 |
| 11. Was the conflict of interest included? | 0 | 0 |
| Total | 8/11 | 9/11 |

0 = No, 1 = Yes
